# Retroperitoneal Laparoscopic Right Heminephrectomy for Renal Cell Carcinoma in a Horseshoe Kidney

**DOI:** 10.1002/iju5.70098

**Published:** 2025-09-23

**Authors:** Yusuke Andoh, Atsuhiko Ochi, Hidetsugu Takahashi, Minoru Nakazono, Akira Komiya, Hiroshi Kuji, Koichiro Suzuki, Naoki Shiga, Takahiro Kimura, Hirokazu Abe

**Affiliations:** ^1^ Department of Urology Kameda Medical Center Kamogawa Chiba Japan; ^2^ Department of Urology The Jikei University School of Medicine Tokyo Japan

**Keywords:** heminephrectomy, horseshoe kidney, laparoscopic surgery, renal cell carcinoma, retroperitoneal approach

## Abstract

**Introduction:**

Laparoscopic heminephrectomy for renal cell carcinoma in horseshoe kidneys has been previously reported, but reports on the retroperitoneal approach remain limited. This paper presents a case demonstrating the feasibility and effectiveness of this surgical technique.

**Case Presentation:**

A 74‐year‐old woman was diagnosed with two renal cell carcinomas in the right side of a horseshoe kidney in contrast‐enhanced computed tomography. A laparoscopic right heminephrectomy was performed using a retroperitoneal approach. This approach allowed for effective management of the complex vasculature supplying the isthmus. Additionally, the inferior mesenteric artery served as a reliable anatomical landmark, facilitating the identification of the optimal site for isthmus transection.

**Conclusion:**

Retroperitoneoscopic heminephrectomy is a safe and effective surgical option for renal cell carcinoma in patients with horseshoe kidneys and represents an anatomically rational approach.

**Abbreviations**
Ao: aorta
CT: computed tomography
HSK: horseshoe kidney
IMA: inferior mesenteric artery
Is: isthmus
IVC: inferior vena cava
RA: retroperitoneal approach
RCC: renal cell carcinoma
RP: renal pelvis
SMA: superior mesenteric artery
TA: transperitoneal approach
Ur: ureter

**Summary**

- Retroperitoneoscopic right heminephrectomy was performed for renal cell carcinoma in a horseshoe kidney.
- Despite complex anatomy due to vascular variation and the isthmus, the retroperitoneal approach allowed safe vascular control and precise isthmus division.

## 1 Introduction

Heminephrectomy is a surgical treatment for malignancies arising in horseshoe kidneys (HSKs), requiring division of the isthmus and management of complex vascular anatomy, as HSKs typically possess approximately twice the number of renal arteries compared to normal kidneys [1]. Recently, laparoscopic techniques, including robot‐assisted procedures [2, 3], have become common for heminephrectomy. Most procedures are performed via the transperitoneal approach (TA), which provides a wider operative field and improved renal mobility intraoperatively [4]. In contrast, the retroperitoneal approach (RA) is less frequently used and is generally reserved for patients with a history of multiple abdominal surgeries or infection‐related complications [4].

Here, we report a case of retroperitoneoscopic heminephrectomy for right‐sided renal cell carcinomas in an HSK, demonstrating the advantages of RA in vascular control and isthmus division.

## 2 Case Presentation

**FIGURE 1:**
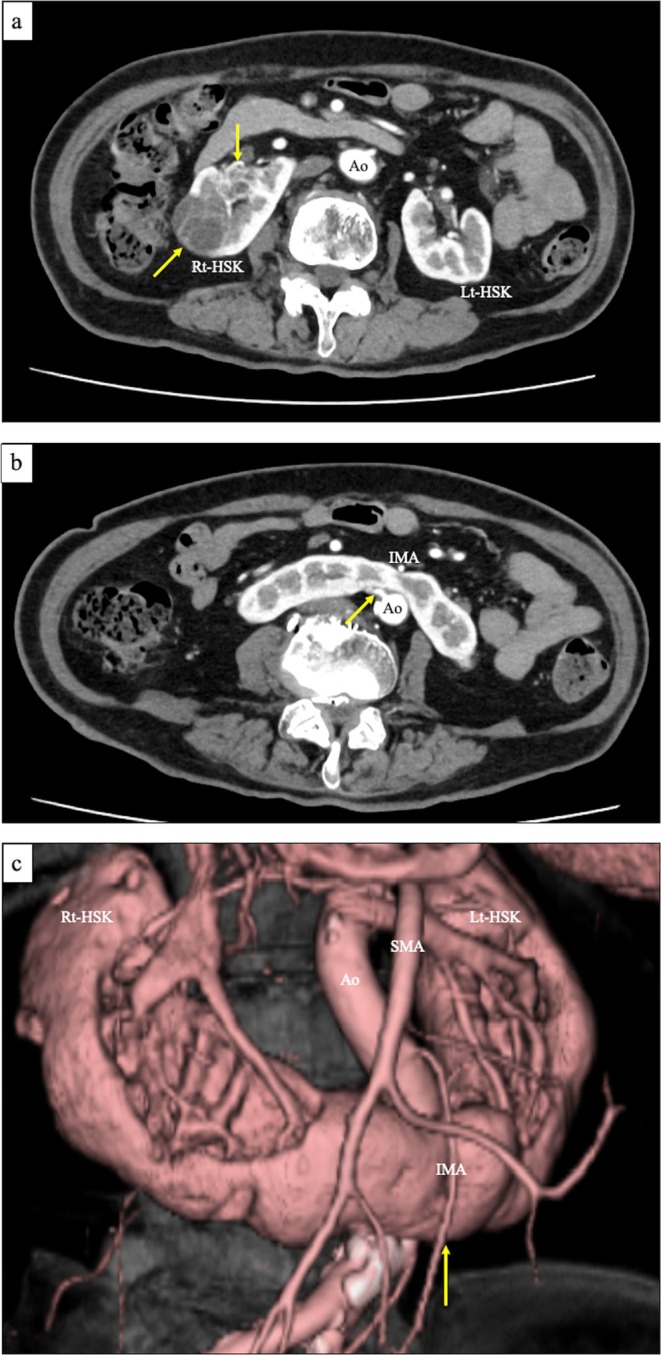
Preoperative contrast‐enhanced computed tomography findings. (a) Two renal tumors located on the right side of the horseshoe kidney (arrows). (b) A renal artery originating from the aorta enters directly into the posterior aspect of the isthmus (arrow). (c) The inferior mesenteric artery crosses the anterior aspect of the isthmus (arrow).

A 74‐year‐old woman (height: 155 cm; weight: 54 kg) with a history of abdominal surgery for uterine myoma was referred to our department after two renal cell carcinomas (RCCs) were identified on the right side of an HSK during a preoperative contrast‐enhanced computed tomography (CT) scan for cholelithiasis. The tumors measured 36 × 33 × 33 mm and 24 × 22 × 19 mm, both containing cystic components (Figure 1a). CT revealed five renal arteries supplying the right side of the HSK: two entering the right renal hilum and three connecting directly to the posterior aspect of the isthmus—two from the aorta (Figure 1b) and one from the right common iliac artery. The isthmus was located posterior to the inferior mesenteric artery (IMA) (Figure 1c). The patient was diagnosed with two right‐sided RCCs without metastases (cT1aN0M0). At that time, robot‐assisted partial nephrectomy was not yet available at our institution. After discussing the risks and potential complications, the patient opted for heminephrectomy. We performed a retroperitoneoscopic right heminephrectomy under general anesthesia with the patient in the left lateral position.

**FIGURE 2:**
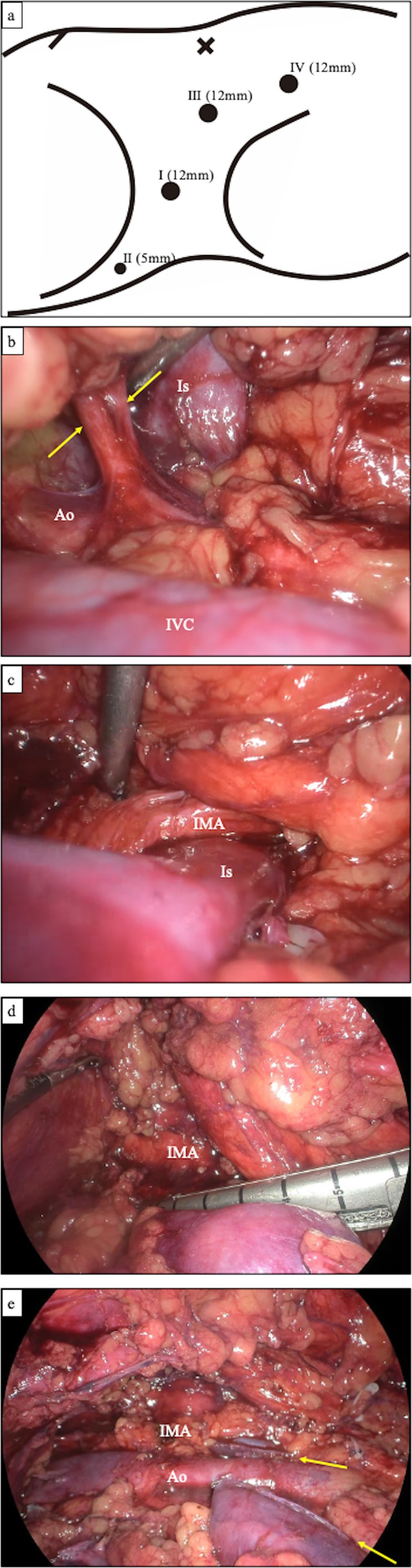
(a) Trocar placement. I: Camera port for retroperitoneoscopy. II: Manipulation port for the surgeon's left hand. III: Manipulation port for the surgeon's right hand. IV: Assistant port. The isthmus was transected using a laparoscopic stapler with 60‐mm staples (Endo GIA Black Reload with Tri‐Staple Technology), which typically requires a 15‐mm trocar. Therefore, trocar no. IV was temporarily removed, and the stapler was inserted directly through the skin incision. (b–e) Retroperitoneoscopic findings. (b) Two renal arteries (arrows) entering directly into the posterior aspect of the isthmus from the aorta. (c) Complete exposure of the isthmus. The inferior mesenteric artery crosses its anterior aspect. (d) Transection of the isthmus using a laparoscopic stapler. (e) Post‐resection view of the isthmus. Arrows indicate the stapler lines.

The retroperitoneal space was created using a dissecting balloon, and four trocars were inserted with a 0‐degree rigid endoscope (Figure 2a). The lateroconal fascia was incised, and the posterior renal fascia was dissected to identify the inferior vena cava and right ureter. The renal arteries entering the isthmus were ligated first (Figure 2b). A small renal vein from the isthmus was identified and divided. The renal arteries entering the right renal hilum were then ligated, and the right ureter was divided. The anterior side of the kidney was dissected along with the anterior renal fascia, and the renal vein was transected using a laparoscopic stapler. Subsequently, the anterior isthmus was dissected, and the IMA was identified. The entire circumference of the isthmus was exposed on the posterior side of the IMA (Figure 2c). A laparoscopic stapler with 60‐mm staples (Endo GIA Black Reload with Tri‐Staple Technology) was inserted through the assistant port incision, and the isthmus was transected (Figure 2d,e). The right adrenal gland was preserved, and the right‐sided HSK was removed via the retroperitoneum. The incisions were then closed. The operative time was 2 h and 14 min, with an estimated blood loss of 5 mL. The postoperative course was uneventful, and the patient was discharged on the fourth postoperative day.

**FIGURE 3:**
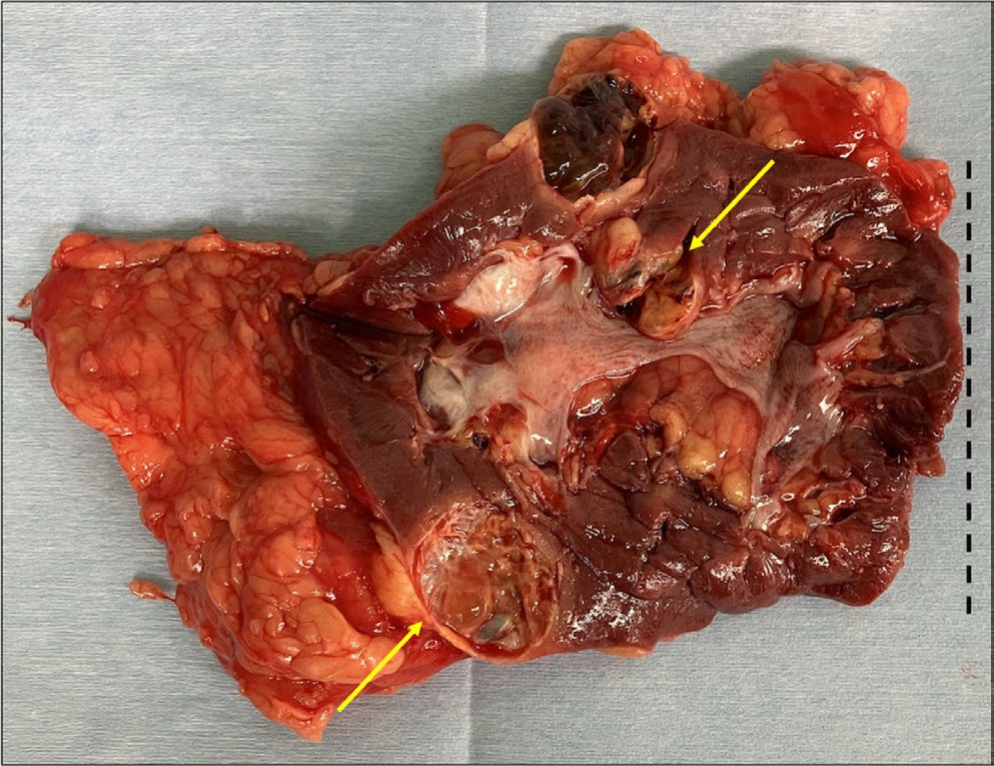
Gross right horseshoe kidney specimen after sectioning and opening. Two tumors are marked with arrows. The dashed line indicates the resection margin of the isthmus. The right renal pelvis and calyces have been completely removed.

**FIGURE 4:**
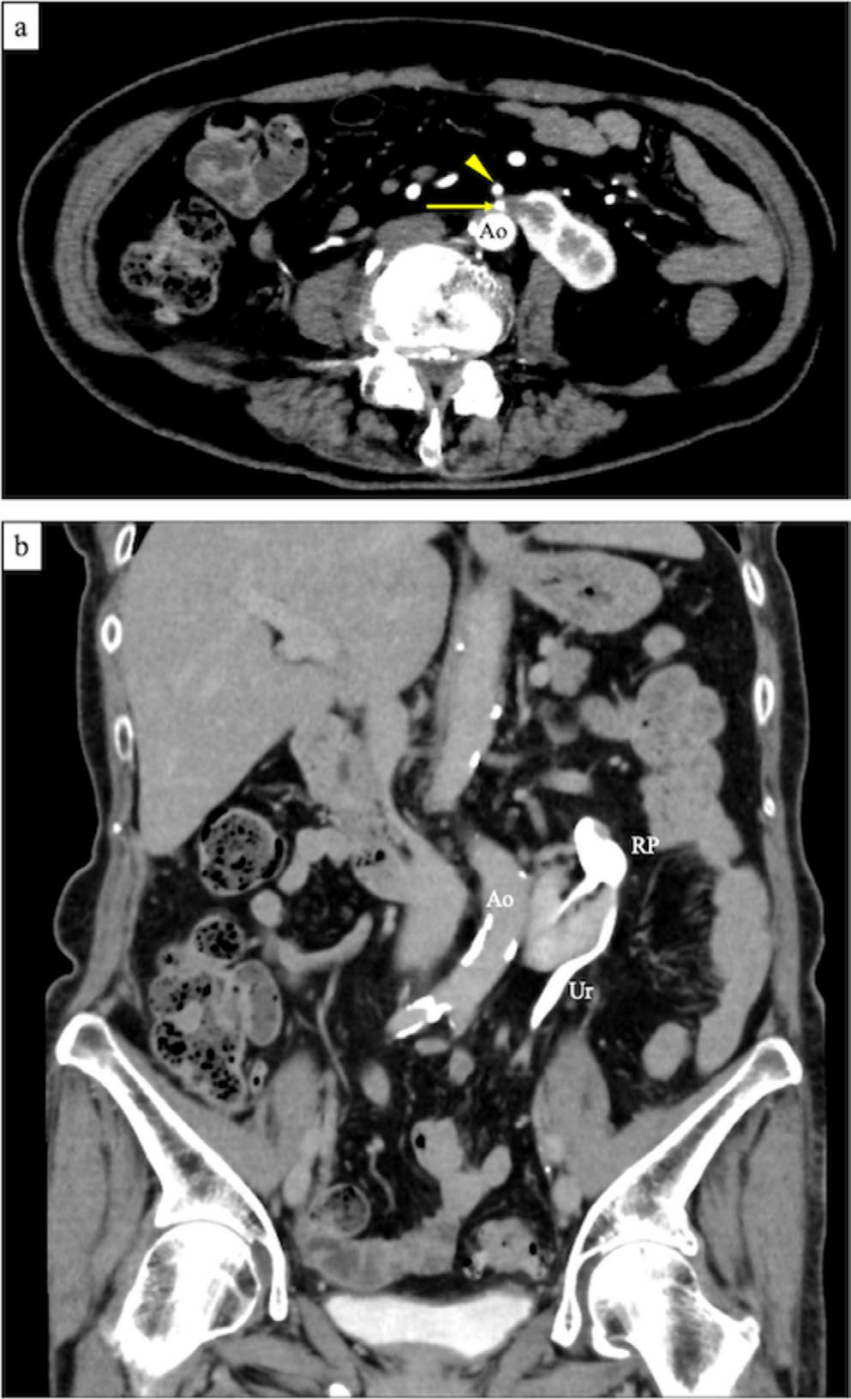
Contrast‐enhanced computed tomography at 3 months postoperatively. (a) The isthmus was resected posterior to the inferior mesenteric artery (arrowhead). The laparoscopic stapler line is indicated by the arrow. (b) Contrast enhancement of the renal pelvis of the left kidney and the left ureter. No evidence of urinoma or residual right renal pelvis/calyces is observed at the remaining isthmus.

Macroscopic examination of the resected right HSK revealed a weight of 210 g, two RCCs, and complete resection of the right renal pelvis and calyx at the isthmus (Figure 3). Pathological analysis confirmed two pT1a clear cell RCCs. Contrast‐enhanced CT at 3 months postoperatively showed no recurrence of renal cancer or residual urinary tract tissue at the isthmus (Figure 4a,b).

## 3 Discussion

In this case, we performed a retroperitoneoscopic heminephrectomy for two RCCs located on the right side of an HSK. The RA facilitated the ligation of complex renal vessels, particularly those at the isthmus. Moreover, the anatomical relationship between the IMA and the isthmus enabled accurate identification and safe dissection of the isthmus.

HSKs typically have a greater number of renal arteries than normal kidneys (4.57 ± 1.39 vs. 2.4 ± 0.43), with a significantly higher proportion of arteries branching caudally from the IMA (43.74% vs. 0.33%) [1]. In this case, we transected three arteries and one vein at the isthmus. These vessels were located posterior to the isthmus, making them easily accessible via RA. Without prior isthmus dissection, such access would have been more challenging using the TA.

Isthmus division is a critical step in heminephrectomy. Approximately 80% of isthmuses contain functional renal parenchyma [5]. Inadequate or improperly positioned division may lead to complications such as urinary fistula [6]. Therefore, accurate identification and precise division of the isthmus are essential. In this case, preoperative CT revealed that the IMA was located anterior to the isthmus, with no renal calyces in that region, indicating a safe site for division. Various techniques for isthmus division have been reported, including monopolar scissors, a laparoscopic stapler, and ultrasonic devices [7, 8, 9, 10]. We utilized a laparoscopic stapler to divide the isthmus just posterior to the IMA, successfully avoiding urinary tract injury. Postoperative CT confirmed the absence of urinary leakage. Notably, the RA provided clear visualization of the anatomical relationship between the IMA and the isthmus, serving as a reliable landmark for determining the optimal division site.

The average operative time for laparoscopic heminephrectomy in adult patients with HSK and RCC is approximately 4 h and 5 min, with no significant difference between TA and RA [10, 11, 12, 13, 14, 15]. For our patient, preoperative confirmation of vessel and isthmus anatomy allowed for safer and more efficient ligation and dissection, contributing to a shorter operative time.

In recent years, robot‐assisted partial nephrectomy has been employed for the treatment of small RCCs in patients with HSK [2]. At our institution, robotic partial nephrectomy had not yet been introduced at the time of this case. Therefore, after thorough informed consent, we proceeded with heminephrectomy. The insights gained from this case may continue to be valuable for future heminephrectomies, especially in situations where partial nephrectomy is not indicated.

In conclusion, retroperitoneoscopic heminephrectomy offers a safe, effective, and minimally invasive surgical option for the treatment of RCC in patients with HSK. This approach provides an anatomically sound and rational technique tailored to the unique vascular and structural characteristics of HSK.

## Ethics Statement

This study has been approved by a suitably constituted Ethics Committee of Kameda Medical Center. The approval number is 24‐039.

## Consent

Informed consent for publication was obtained from the patient.

## Conflicts of Interest

The authors declare no conflicts of interest.
